# Determinants of cancer care pathways at Wajir County, Kenya: patient perspectives

**DOI:** 10.3332/ecancer.2025.1841

**Published:** 2025-02-07

**Authors:** Fatuma Affey, Dabo Galgalo Halake, Grace Muira Wainaina, Hussein Ali Osman, James G Ndukui, Houda Abdourahman, Omar Abdihamid

**Affiliations:** 1 Department of Nursing & Midwifery, Umma University, PO Box 713 Kajiado, Kenya; 2 Department of Pathology, Hopital De Balbala Cheiko, PO Box 669, Balbala, Republic of Djibouti; 3 Garissa Regional Cancer Center, Garissa County Referral Hospital, PO Box 29-70100, Garissa, Kenya; 4 Division of Cancer Care and Epidemiology, Queen's Cancer Research Institute, Queen's University, Kingston, Canada

**Keywords:** cancer care pathways, mixed-method, patient perspectives, Wajir County

## Abstract

**Background:**

Cancer represents a major public health issue with substantial morbidity and mortality in low-resource settings such as Kenya. This study focuses on Wajir County in northern Kenya, a region with limited cancer care infrastructure and high unmet needs. Despite recent efforts to decentralize cancer care in Kenya, including establishing regional cancer centres in Garissa, Nakuru, and Mombasa, access to screening, diagnostics, and treatment remains constrained, particularly in rural areas. The absence of comprehensive cancer care pathways and a specialized oncology workforce in Wajir County exacerbates challenges in early detection, treatment, and palliative care. The study evaluated the availability of cancer care services at Wajir County Referral Hospital (WCRH), including screening, diagnostic services, treatment modalities, and referral systems. The study further explores the gaps in cancer care, focusing on patient perspectives, and proposes potential solutions to address these challenges.

**Methods:**

This study used mixed-methods (qualitative and quantitative) methods to understand cancer care from the perspective of patients at WCRH. It involved adult patients (over 18) with a confirmed cancer diagnosis who were receiving treatment or follow-up care between February and April 2024. Data were gathered through interviews and surveys, with research assistants helping with language translation and community navigation. The study collected information on demographics, cancer types, and prevalence rates, which were analysed using descriptive statistics. The qualitative data focused on patients' experiences with cancer awareness, treatment, and care gaps, and were analysed for common themes. Ethical approval was obtained, and informed consent was given by all participants.

**Results:**

This study involved 25 cancer patients (12 males, 13 females) receiving treatment at WCRH. The most common cancers were esophageal (44%), cervical (28%), breast (24%), and prostate (8%). Delays in diagnosis were significant, with 12% of patients waiting over 6 years, 24% waiting 4–6 years, and 40% waiting 1–3 years before seeking care. Most diagnoses were made at WCRH (64%), with others diagnosed at the Garissa Cancer Centre (22%) or in Nairobi (20%). Diagnostic tools available at WCRH included pap smears, mammograms, PSA tests, ultrasound, CT scans, and biopsies. However, access to these tools was limited, with barium swallow (32%) being the most frequently used for esophageal cancer, followed by pap smears, biopsies, and ultrasound (16% each). Patient awareness of cancer screening was higher for cervical (68%) and breast cancer (60%) but lower for prostate cancer (32%) and esophageal cancer (4%). Despite awareness, only 8% had previously undergone screening. Regarding treatment, most patients (80%) were aware of surgical options, while fewer knew about chemotherapy (28%) or palliative care (12%). When treatment was unavailable at WCRH, most patients preferred the Garissa Cancer Centre (80%) or Nairobi (52%). Financial challenges were the primary barrier to treatment for 88% of patients, and patients suggested improving local cancer care, subsidizing treatment, and enhancing early detection and screening services.

**Conclusion:**

The findings indicate a high burden of late-stage cancer diagnoses, insufficient cancer screening and treatment services, and limited access to cancer care pathways and patient navigation systems. These results underscore the urgent need for improved cancer care pathways, enhanced awareness, and increased healthcare capacity to reduce cancer morbidity and mortality in northern Kenya. This study contributes to understanding the cancer care landscape in Wajir County and provides a foundation for future health policy initiatives aimed at bridging existing gaps in cancer care.

## Introduction

In 2024, the latest global cancer statistics depicted cancer as a major societal, public health and economic problem in the 21st century. It causes 3 in 10 global premature deaths from non-communicable diseases (30.3% in those aged 30–69 years), and it is among the three leading causes of death in 177 of 183 countries in this age group [1].

In Kenya, the latest 2022 Globocan data shows 44,726 cancer cases diagnosed with 29,317 deaths. The leading cancers in incidence and mortality among Kenyan women include breast, cervix and esophageal cancers (ECs), while in men, prostate, esophagus and colorectum are the most common and leading causes of cancer-related mortality in that order [2].

Challenges in cancer care in Kenya include lack of cancer awareness and patient education, poor health-seeking behavior often contributing to late diagnosis, cancer diagnosis-related stigma, taboos, cultural barriers to seeking treatment and very few oncology-allied professionals [3, 4]. Access to health services such as early detection, especially in breast cancer as recommended by the World Health Organisation’s breast cancer initiative implementation framework is particularly low for over 70% of urban Kenyans living in informal settlements where health outcomes are dire. There are stark differences in morbidity and mortality rates in these areas compared to other parts of the country, aggravated by low socioeconomic burden, inadequate public health delivery and extensive inefficiencies [5, 6]. The cancer scenario in rural areas poses health challenges marked by disparities such as sparse health facility distribution and financial constraints hindering access to private healthcare [7].

Cancer care in Kenya is not publicly funded. Patients co-pay with insurance and out of pocket, leading to catastrophic spending among patients and their families, exposing the existing inequity in cancer care costs among poor patients [4]. Moreover, cancer treatment access is limited for most Kenyans, with all major cancer centres predominantly domiciled in the capital city [8]. However, there have been recent efforts by the Ministry of Health in Kenya to decentralise cancer care, leading to the launch of three regional cancer centres: Garissa, Nakuru and Mombasa regional cancer centres [9].

Garissa Regional Cancer Centre has heralded hope for cancer patients in the northern Kenya region, as for more than 60 years there has not been a cancer centre in northern Kenya, rendering cancer patients in Mandera, Wajir and Garissa counties to grapple with huge unmet needs in cancer care [10]. However, data on the cancer burden in these countries are scarce. Notably, a health facility-based retrospective review in Wajir County revealed that the most common cancers were EC (69.3%), followed by stomach (10.4%), prostate cancer (8.6%), cervical (3.5%) and liver cancer (1.1%) between 2014 and 2019 [11].

Moreover, the findings from the Kenya Health Facility Assessment showed that cancer screening services are only available in less than a third (26%) of health facilities in northern Kenya, while 2% of the facilities have cancer treatment services [12]. The lack of these cancer screening services translates to a high proportion of cancer cases diagnosed in the late stages, which further contributes to poor prognosis.

The Kenya Demographic Health Survey [13] also showed that only 5% of women in northern counties had heard about cervical cancer, with only 0.4% going for screening. In terms of breast cancer, 2.2% of the women in northern counties had performed self-breast examinations, with only 1.4% of them having been examined by a healthcare provider [14]. This report also highlighted the lack of awareness about prostate cancer, with only 22.6% of men who had heard about prostate cancer; none of them underwent any screening.

Most cancer screening services are available in urban areas as compared to rural settings in Kenya [12]; therefore, the true burden of cancer in rural settings is underestimated, given the insufficiency of cancer registries and surveillance networks [14].

In Northern Kenya, cancer patients travel long distances to seek care especially pain and palliative care services [15]. In such settings, simple and affordable cancer diagnostic tools are required for cancer screening. The availability and accessibility of cancer screening services in Northern Kenya, particularly in Wajir County, are unknown. Establishing the availability of cancer screening services in this county is paramount so that the true burden of cancer can be established.

Wajir County also lacks an oncology workforce with only one oncologist and a limited number of oncology nurses. There is also a lack of other essential oncology-allied personnel such as oncology counselors, oncology nutritionists and palliative care nurses. Radiotherapy treatment is not offered in Wajir County, albeit there is some form of chemotherapy services available at the Wajir County Referral Hospital (WCRH). Most cancer cases are referred to nearby Garissa regional cancer centres, where such services are available from 2021 [10].

Cancer care is more sophisticated and requires a multidisciplinary approach to improve patient outcomes and increase survival rates [16]. Cancer care pathways (CCPs) are among the new models under evaluation for providing evidence-based oncology care [17]. CCPs call for the utilisation of care pathways that offer patient-centreed care and improve the quality of cancer care. Due to the paucity of CCPs and their limited capacity in terms of accessibility among cancer patients in northern Kenya. A study on cancer-related pain at the Garissa County Referral Hospital revealed a high cancer pain prevalence of 78%, with most patients experiencing moderate to severe pain coupled with suboptimal cancer pain management [18, 19]. It is upon this basis that this study was carried out with two main goals. First to establish the prevalence of cancer in the region, and second, to assess the availability of cancer care pathways at WCRH including cancer screening and diagnostic services, cancer treatment modalities, referral pathways in place and existing cancer care challenges and alternative solutions available in WCRH. To the best of our knowledge, this is the first study in Wajir to assess cancer care gaps and unmet cancer care needs from patients’ perspectives.

## Methodology

Using a mixed method (qualitative and quantitative approaches), this study sought to understand the CCP from patients’ perspectives at the WCRH. Patients were interviewed on the thematic areas of cancer awareness, availability of cancer screening services, treatment options available at WCRH and specific cancer care gaps.

Adult patients (aged >18 years) with a confirmed cancer diagnosis over 3 months (February to April 2024), who were receiving treatment or were followed up at the WCRH were recruited. Patients' records were utilised to determine the cancer prevalence in the region. Children (<18 years), patients with an unconfirmed cancer diagnosis, and those who were severely ill to consent to study inclusion were also excluded. Data were collected from cancer patients using a semi-structured questionnaire and interview guide, and research assistants were utilised for language translation, navigation within the community geography and social-cultural dynamics. The study was approved by the ethics review board of the University of Eastern Africa, Baraton (Ethics approval number: 815298) and research permit was obtained from NACOSTI (NACOSTI/P/24/37193). Prior to data collection, the patients were informed about the study and written informed consent was obtained from the patients.

Descriptive data analysis was conducted using the Statistical Package for the Social Sciences version 24. Descriptive data collected included demographic characteristics (age, sex, marital status, income level and education level), types of cancer patients were diagnosed with and prevalence rate (expressed in frequencies and percentages). Thematic analysis was used to analyze qualitative data on challenges to cancer treatment and preferred solutions to cancer care gaps within the region

### Patient involvement

All study participants were consented consecutively and informed about the purpose of the study and were also central in the dissemination of CCPs information

## Results

**Figure 1.**
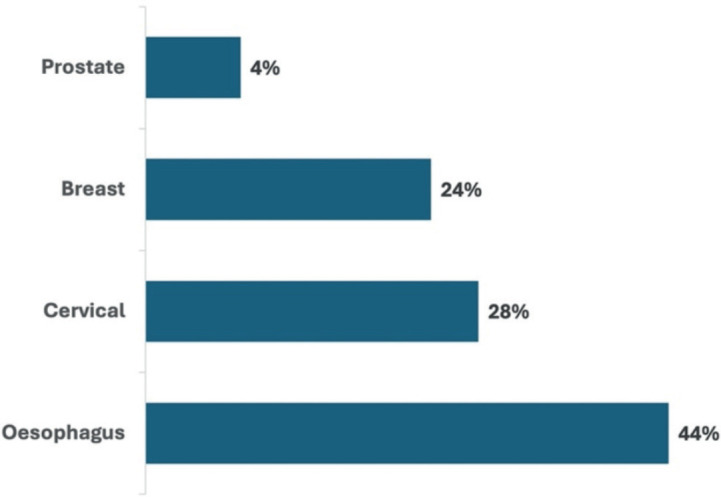
Types of cancer diagnosed at WCRH.

**Figure 2.**
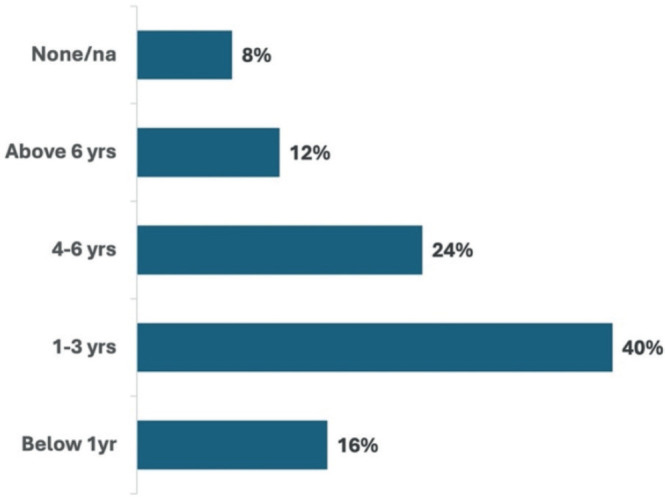
Time to first diagnosis in years.

This study enrolled 25 patients (12 male, female 13 women) with a confirmed cancer diagnosis at the time of follow-up and receiving treatment at WCRH. The distribution of cancer subtypes included EC (44%), cervical cancer (28%), breast cancer (24%) and prostate cancer (8%) (Figure 1). Patients mentioned obvious delayed cancer diagnosis with 12% of the patients waiting over 6 years, 24% waiting over 4 to 6 years and 40% waiting over 1 to 3 years before presenting to a facility for a diagnosis as shown in Figure 2.

Regarding the facility of initial diagnosis, (64%) were diagnosed at the WCRH, 4% at the mother and child department, 22% at the Garissa Cancer Centre and 20% in the capital Nairobi, which is 680 km away.

Cancer diagnostic tools available at WCRH include pap smears, mammograms, prostate-specific antigen (PSA), barium swallow, ultrasound, computed topography (CT) scans, endoscopy, X-rays and biopsy. The percentages of patients with access to each of these diagnostic tools were as follows: barium swallow for EC at 32%, ultrasound, biopsies and Pap smears all had a similar utility of 16%), CT scan (12%), mammograms (8%), endoscopy (8%) and X-rays at 4%.

Assessment of knowledge regarding the availability of cancer screening services at WCRH, most patients were aware of the availability of cervical cancer screening (68% knew about pap smear), followed by breast cancer at 60% (mammogram), 32% for prostate cancer (PSA) and barium swallow (4%). However, at the time of this study, only 8% of patients had undergone prior cancer screening.

When asked about the availability of cancer treatment modalities available at WCRH, 80% of the patients mentioned surgery, 28% knew about chemotherapy, hormonal therapy (8%) and palliative care services (12%) and 4% did not know about the availability of any cancer treatment.

Most of the patients (80%) preferred to go to Garissa Regional Cancer Centre, 52% opted for Nairobi for treatment if such treatment was not available at WCRH, and 4% preferred home-based care. As expected, 88% of the patients cited financial toxicity as the main challenge to cancer treatment, followed by treatment access. The patients suggested establishing a local cancer centre (32%) and subsidising the cost of cancer treatment (24%), early screening (28%), early diagnosis (16%), patient education (12%), early referral (8%) and access to health insurance (4%).

## Discussion

The state of cancer care access in Kenya continues to be heterogeneous concerning inequity in access to quality care, income brackets, education level, health-seeking behaviors, culture, cost affordability, health insurance status and uneven distribution of cancer centres [4].

CCPs call for the utilisation of pathways that offer patient-centred care and improve the quality of cancer care [17]. Cancer care access in Kenya is a kin to accessing a ‘maze’ as one author quipped [20]. This is especially true for patients in rural counties in Kenya such as Wajir County. Accessing cancer screening and treatment services is one of the major hurdles due to concentrations of essential cancer care services within a 5-km radius of each other in the capital Nairobi [8]. Consequently, the lack of CCPs in Wajir County and the increasing burden of cancer among the northern Kenyan communities prompted the authors to carry out this study at the WCRH.

Our study highlights often-forgotten patient-reported outcomes by focusing on patient perspectives. Largely, a nomadic population of ethnic Somali, the communities in this region grapple with an increasing burden of cancer. Compounded by low literacy level and low socioeconomic status (only 12% earning 269 $ per month), as shown in Table 1. Poverty and family income bracket are a key determinant of health-seeking behavior and access to quality care [21].

The present study found that the leading cancer at the WCRH was EC at 44%, followed by cervical cancer (28%), breast cancer (24%) and prostate cancer (4%). The incidence of EC has been reported to be on the rise in northern Kenya. A recent study by Abdihamid *et al* [22] showed the patterns of EC presentation, including late disease presentation, suboptimal cancer staging, loss of follow-up, low uptake of healthcare insurance and cultural barriers as the main drivers of poor outcomes in EC cancer among the communities in this region.

Delayed cancer diagnosis and late presentation are the main drivers of cancer-related mortality and morbidity in sub-Sahara Africa with nearly 2,000 lives being lost daily in Africa alone. The major challenge for cancer care in Africa is equity and prioritisation, as cancer is not receiving adequate attention from policymakers and strategic stakeholders in the healthcare space [23].

Similarly, our study showed patients had as long as 6 years before they got diagnosed with cancer, mostly associated with lack of education and poor access to cancer care. Daniel *et al* [24] have also shown that financial constraints, lack of patient breast cancer awareness and healthcare practitioner misdiagnosis as the main drivers of delayed diagnosis and treatment initiation in breast cancer patients in a major referral hospital in Kenya. Mwenda *et al* [25] also showed cost of care and quality of clinical evaluation as predictors of late-stage presentation in patients with cervical cancer in Kenya.

Despite the awareness of the availability of cancer screening services at the WCRH, only 8% of our study cohort had prior voluntary cancer screening, citing the lack of community education on cancer awareness. There is generally a low uptake of cancer screening services in Kenya, with the Kenya Cancer Policy Report 2019–2030 showing only 16.4% of eligible Kenyan women have ever been screened for cervical cancer. The report also shows less than 20% of health facilities in Kenya are screening for cervical cancer, and only 1% of eligible women were screened for breast cancer using mammography in 2018/19 despite the availability of mammography equipment in most county referral hospitals [26].

While patients were aware of the existing cancer treatment such as surgery at WCRH, many patients eventually went to the neighboring Garissa Regional Cancer Centre [10], and the capital Nairobi, understandably due to the availability of chemotherapy and radiotherapy at the GRCC and Nairobi. Therefore, establishing collaboration between county health facilities and nearby cancer centres is urgently needed for timely referral, thus improving patient outcomes.

Cancer-related cost is a global phenomenon and a leading barrier to cancer treatment and is estimated at 255.2 trillion [27]. In our cohort, 88% of patients mentioned the high cost of cancer treatment when propped about the challenges faced by cancer patients at WCRH. The patients further suggested access to care that access as the second most common challenge and recommended setting up a local cancer centre, subsidising the cost of cancer treatment, improving early screening and diagnosis and access to health insurance, as the main solutions to overcome these challenges.

It is, therefore, important that the country develops a strategic framework that will guide all levels of governance to effectively plan and implement cancer prevention and control interventions. This strategy will thus provide a roadmap to inform strategic measures in cancer prevention and control at both levels of government as well as among all stakeholders (Table 2).

This study had several strengths and limitations. The present study showed that it is equally important to consider patient perspectives in addition to healthcare perspectives when formulating CCPs in hospitals, especially in resource-constrained settings. The study also highlights the existing gaps in cancer care in Wajir County, reaffirming the increasing incidence of EC in the region; hence, the need to invest in diagnostic services such as endoscopy and the need to educate communities using community health outreach programs on the availability of cancer screening services available at WCRH. The limitations of this study include its qualitative nature, making it susceptible to recall biases, small sample size, inability to derive causality and lack of statistical representation. Nonetheless, the overall aim of the authors was to highlight the patients’ perspectives about their cancer care, which is in and of itself a worthy patient advocacy duty.

## Conclusion and future recommendations

Cancer is a burden in all parts of Kenya. In our study, EC was the most prevalent cancer in Wajir County, with huge gaps in CCPs, low uptake of available essential cancer screening services due to lack of awareness and a high burden of cancer-related costs. Most patients preferentially recommend decentralising cancer centres. Consequently, there is a need for both levels of government to collaborate in improving CCPs with a focus on early detection, screening, public education on cancer and establishing a cost-effective health package for cancer screening, diagnosis and treatment.

## Conflicts of interest

None to disclose.

## Funding

None to declare.

## Figures and Tables

**Table 1.** Social demographic of cancer patients at Wajir County Hospital.

Characteristics	Numbers	Absolute numbers	Percentages (%)
Gender	Male Female	12 13	48 52
Age distribution (years)	18–30 years 31–40 years 41–50 years 51–64 years	7 14 3 1	28 56 12 4
Ethnicity	Somali	19	76
	Non-Somali	6	24
Education level	Primary	5	20
	Secondary	8	32
	Tertiary	8	32
	No-formal education	4	16
Monthly income	Less than Ksh 5,000 (38$)	4	16
	Ksh 6,000–10,000 (46$–76$)	9	36
	Ksh 10,000–15,000 (46$–115$)	4	16
	Ksh 15,000–20,000 (46$–153$)	4	14
	Ksh 25,000–30,000 (192$–230$)	1	4
	Above Ksh 35,000 ($269)	3	12
	Key: Ksh (Kenyan shilling) $: Dollar		

**Table 2.** Proposed solutions to existing cancer care gaps at WCRH.

1. Cancer research: There is an urgent need to carry out epidemiological and etiological cancer studies to understand why EC is the most prevalent in Wajir County and its associated risk factors
2. Data generation: There is a need to improve record keeping of patients’ data and digital migration
3. Cancer care fund: There is an urgent need to establish an essential health package that is cost-effective and feasible for cancer patients and their families to avoid eminent catastrophic spending and financial toxicity
4. Public cancer awareness: There is an urgent need to upscale cancer education, the importance of symptom reporting, and information on regional cancer centres
5. Equity in cancer access: There is a need to ensure equitable access to the entire range of cancer care continuum with a focus on the most vulnerable populations.
